# Elevated CO_2_ concentration induces changes in plant growth, transcriptome, and antioxidant activity in fennel (*Foeniculum vulgare* Mill.)

**DOI:** 10.3389/fpls.2022.1067713

**Published:** 2022-12-09

**Authors:** Na-Yeon Jo, Junkyung Lee, Ji-Eun Byeon, Hong-Jin Park, Jong-Won Ryoo, Sun-Goo Hwang

**Affiliations:** ^1^ College of Life and Environment Science, Sangji University, Wonju-si, South Korea; ^2^ Department of computer and Engineering, Sangji University, Wonju-si, South Korea

**Keywords:** elevated CO2, transcriptome, fennel, antioxidant properties, plant growth

## Abstract

**Introduction:**

Fennel (Foeniculum vulgare Mill.) is widely used to produce natural bio-materials. Elevated CO_2_ (eCO_2_) concentrations in the atmosphere improve the net photosynthesis of plants.

**Methods:**

The aim of the present study was to investigate distinct changes in fennel growth characteristics and phytonutrient contents under different CO_2_ concentrations. The effects of 400 and 800 ppm concentrations on plant growth and antioxidant activity were observed under hydroponics.

**Results and Discussion:**

Plant growth was improved by eCO_2_ concentrations. We also observed diverse changes in nutrient solution (pH, electrical conductivity, and dissolved oxygen) and environmental factors (temperature and humidity) in greenhouse under light or dark conditions. Electrical conductivity increased under dark and eCO_2_ conditions, whereas the pH decreased. Additionally, we performed transcriptome analysis and identified CO_2_-responsive differentially expressed genes. In the 800 ppm group, genes involved in photosynthesis and Karrikin response were upregulated whereas those involved in syncytium formation were downregulated. Four upregulated differentially expressed genes involved in flavonoid biosynthesis and total flavonoid content were relatively increased under the 800 ppm CO_2_ condition. In contrast, antioxidant activity, including total phenolic content, scavenging activity, ferric ion reducing antioxidant power, and reducing power were decreased in fennel under relatively high eCO_2_ concentrations. Moreover, different light intensities of 12 or 24 lx did not affect the growth and antioxidant activity of fennel, suggesting eCO_2_ has a stronger effect on plant improvement than light intensity. The results of the present study enhance our understanding of the positive effects of CO_2_ on the growth and antioxidant activity of fennel.

## Introduction

The concentrations of carbon dioxide (CO_2_) in the atmosphere are rising rapidly owing to the use of fossil fuels and forest destruction following the industrial revolution; in addition, CO_2_ emissions due to industrial fossil fuel combustion are a major cause of global warming (Oh et al., 2016). According to the Intergovernmental Panel on Climate Change (IPCC), the average global temperature and CO_2_ concentration have increased by 0.8°C and 68.9 ppm, respectively, from 1984 to 2020, due to global warming (IPCC, 2022). Furthermore, the average global temperature will rise by 4.7°C by 2100, and the projected CO_2_ concentration is 940 ppm (NIMS, 2022).

The levels of CO2 in the atmosphere have been increasing continuously since the 1960s. According to the National Oceanic and Atmospheric Administration (NOAA)’s Global Monitoring Lab analysis, the atmospheric levels of CO_2_ peaked at approximately 414 ppm in 2021, and was 316.91 ppm in 1960 (NOAA, 2022). Agriculture contributes significantly to anthropogenic global warming, and the reduction of agricultural emissions could play an important role in mitigating climate change. However, it is extremely challenging to meet the growing demands for food, energy, and water while mitigating climate change.

Despite causing global warming, CO_2_ enrichment has been demonstrated to improve crop yield in agricultural greenhouses (Dion et al., 2011). In addition to causing plants to partially close their stomata, increasing CO_2_ concentrations enhance photosynthetic efficiency (Huang et al., 2007), thereby reducing water loss and increasing carbon acquisition efficiency from water (Huang et al., 2007). Therefore, plants exhibit physiological responses, such as improved photosynthesis and stomatal conductance, in response to elevated CO_2_ (eCO_2_) concentrations. Additionally, CO_2_ concentrations influence the contents of plant phenolic compounds, including tannins (Ainsworth and Rogers, 2007; Akula and Ravishankar, 2011). Plant phenolic compounds have several clinical benefits, such as preventing and alleviating symptoms of allergy, arteriosclerosis, inflammation, and oxidative stress. Owing to the extended life expectancy of humans, there is an increasing interest in natural products, including phenols and flavonoids (Badgujar et al., 2014; Hwang et al., 2015).

Environmental factors, such as light, CO_2_ concentration, temperature, and humidity, can cause diverse changes in plant development. Particularly, light intensity and CO_2_ concentrations are important as they serve as energy source and carbon substrate, respectively, for plant growth and carbon assimilation (Muneer et al., 2014; Pan et al., 2020). Studies have demonstrated that plant growth was improved in terms of dry matter accumulation, carbon assimilation, and photosynthetic pigment concentration by the combined effect of eCO_2_ and light intensity (Proietti et al., 2013; Naing et al., 2016; Pan et al., 2020). In plants, eCO_2_ has been reported to increase the light saturation point, resulting in increased efficiency of light use, indicating improved carbon fixation and photosynthetic light capture by eCO_2_ (Faralli et al., 2016). Excessive light intensity has been observed to influence reactive oxidative species, leading to an increase in total phenol (Dey and Harborne, 1997; Edreva, 2005; Oh et al., 2009). The intensity and quality of light has been shown to have diverse impacts on the regulation of plant growth. For example, suitable light intensities led to increase in the fresh weight, dry weight, and stem diameter of tomatoes (Fan et al., 2013). Furthermore, different light intensities led to changes in plant growth, chlorophyll content, and biomass of tomatoes (Fan et al., 2013).

Fennel *(Foeniculum vulgare* Mill.) is a perennial herb of the family Apiaceae that is cultivated in the Mediterranean area for food and its medicinal properties (Oktay et al., 2003). Currently, it is cultivated in many other areas, including Korea (Lee and Kim, 2020). Fennel is used as a food ingredient in salads, spices, alcoholic drinks, and tea, as well as a medicine, as a carminative, diuretic, anti-inflammatory, anti-microbial, and galactagogue (Mahfouz and Sharaf-Eldin, 2007; Malhotra, 2012). The major components of fennel are trans-anethole, estragole, fenchone, and limonene (Lee and Kim, 2020). Trans-anethole, the main phenolic compound in fennel oil, is associated with the prevention of cardiovascular diseases, cancer, and oxidative-stress induced inflammation (Badgujar et al., 2014; Lee and Kim, 2020). In addition, the phenolic glycoside, estragole, exhibits disinfectant and antiseptic properties, and fenchone exhibits antibiotic and antifungal activities (Lee and Kim, 2020).

Fennel seed extract and essential oil exhibit inhibitory effects on bacteria, oxidative stress, and browning (Oktay et al., 2003; Oktay et al., 2003; Roby et al., 2013; Roby et al., 2013; Lee and Kim, 2020; Lee and Kim, 2020). However, no study has investigated the potential effects of eCO_2_ on fennel. The rapidly increasing atmospheric CO_2_ concentrations will have diverse effects on agricultural species. In the present study, we investigated whether eCO_2_ could affect the transcriptome of fennel. We surveyed plant growth, transcriptome, and antioxidant activity under eCO_2_ under hydroponics. We hypothesized that eCO_2_ will alter antioxidant activity *via* changes in the transcriptome in biomass-related metabolic pathways. Although eCO_2_ concentrations and light are known for their positive effect on light utilization efficiency, examining the distinct effect of the carbon substrate on plant growth in comparison with light intensity is necessary. Thus, we attempted to compare the distinct effects of eCO_2_ and light intensity on plant growth and antioxidant activities.

## Materials and methods

### Plant materials and growth conditions

Fennel (*Foeniculum vulgare* Mill.) seeds provided by Asia Seed Company (Asia Seed Co., Seoul, Korea) were germinated in a growth chamber under dark conditions for two weeks at the Sangji University at a mean temperature of 20 °C and 70% humidity. The seedlings were transplanted into rock wool cubes (40 × 40 × 40 mm) for hydroponics, and the plants were grown in a plastic film covered greenhouse (150 × 300 × 150 mm) using a deep flow technique with a mean temperature of 27 °C, mean humidity of 80%, and 12/12-h light/dark photoperiod. We used LED modules (MSIP-REI-3SI-SIF-300, OSRAM, Seoul, Korea) as light sources in the greenhouse. CO_2_ was supplied to each greenhouse at concentrations of 400 ppm or 800 ppm. We observed changes in CO_2_ temperature and humidity in the plants under the different CO_2_ conditions. We measured the temperature and humidity of the greenhouse using an Arduino AM2302 sensor (**Figure S1A**). CO_2_ was measured using a CM1107 as dual beam non-dispersive infra-red detector from CUBIC (Wuhan, China). The CO_2_ sensor is able to measure CO_2_ concentrations in the 0–5,000 ppm range, with an error of ± 30 ppm (**Figure S1A**). A 20-L aluminum cylinder tank with a gas regulator was used to supply CO_2_, which had a pressure gauge inside the bottle, and a bubbler counter and a power adapter (**Figure S1B**). A constant CO_2_ concentration of 400 ppm or 800 ppm was maintained using an Arduino UNO R2 kit with an ATMEGA4809 microcontroller (Microchip Technology Inc., Shanghai, China), and the constant CO_2_ was supplied both day and night. The environmental data detected using the Arduino sensors were transmitted to an IBM 3500 computer in real time at one-minute intervals. The nutrient solution was used in the hydroponics systems at weekly intervals according to previous studies (An and Lee, 1991; Kim and Na, 2018). The nutrient solution comprised 606 mg KNO_3_, 115 mg NH_4_H_2_PO_4_, 236 mg Ca (NO_3_)_2_·4H_2_O, 246 mg MgSO_4_·7H_2_O, 22.6 mg Fe-EDTA, 2.9 mg H_3_BO_3_, 1.8 mg MnSO_4_·4H_2_O, 0.2 mg ZnSO_4_·7H_2_O, 0.08 mg CuSO_4_·5H_2_O, and 0.03 mg NH_4_M_0_O_4_·2H_2_O. All chemical components of the nutrient solution were obtained from DAEJUNG CHEMICAL and METALS company (Gyeonggi-do, Korea). To observe plant growth under different light intensities, fennel was grown in the greenhouses under different light intensities of 12 lx and 24 lx with a mean temperature of 26 °C, mean humidity of 30%, and 12/12-light/dark photoperiod. The light intensity was adjusted in the dark greenhouse by controlling the number of the LED modules. To confirm the experimental light intensities, we measured the light intensities using a DT-92 mini light meter (CEM Co., Shenzhen, China). Humidity and temperature were measured in the greenhouses using an AM2302 sensor (Aosong Electronic Co., Guangzhou, China), which has a measuring error of ± 2% humidity, *via* a polymer humidity capacitor. To observe the nutrient solution changes, we measured the electrical conductivity (EC), pH, and dissolved oxygen (DO) by Lutron WA-2017SD Multi Water Quality Meter (Lutron electronic enterprise Co., Coopersburg, PA, USA).

### Plant growth survey

To compare plant growth under different CO_2_ conditions, we observed plant length, stem diameter, number of tillers, SPAD, and above-ground weight in fennels cultivated under CO_2_ concentrations of 400 or 800 ppm five weeks after planting. Plant length was measured from the rock wool cube to the top part, and stem diameter was measured using Vernier calipers. The SPAD value was measured using a SPAD meter (SPAD-502plus, Minolta, Japan). All measurements were performed in triplicates. The above-ground weights were measured using the leaf and stem tissues excluding roots, before and after dehydration, using a drying machine at 60°C for 24 h.

### *De novo* transcriptome analysis

RNA was extracted from leaf tissues subjected to 400 and 800 ppm CO_2_ concentrations using the Accuprep^®^ Universal RNA Extraction Kit with three replicates. RNA quality was estimated using a spectrophotometer (NanoDrop-nabi, MicroDigital, Seongnam-si, Korea). The RNA extracted from the six samples were subjected to library preparation according to the manufacturer’s instructions, and then sequenced using Illumina NovaSeq 6000 (Illumina Inc., San Diego, CA, USA) with paired-end reading. To generate the genome information of fennels, we used merged raw RNA sequencing data for *de novo* transcriptome assembly using Trinity assembler (https://github.com/trinityrnaseq/trinityrnaseq). The assembled contigs were used to find transcripts, including open reading frames over 100 amino acids, using Trans Decoder (https://github.com/TransDecoder/TransDecoder). The annotation of long open reading frames was performed by BLAST (Altschul et al., 1990) using UniProtKB/Swiss-Prot data (https://www.ebi.ac.uk/uniprot/download-center). The redundant transcripts were removed using the CD-hit program (Li and Godzik, 2006). Gene expression levels were estimated using the Trinity Perl script, and then the differentially expressed genes (DEGs) between plants under 400 ppm and 800 ppm CO_2_ conditions were identified using the edgeR method (Robinson et al., 2010) with a threshold false discovery rate (FDR) of 0.05. Functional enrichment analysis (FDR< 0.05) of DEGs among the total genes detected in fennels was conducted using DAVID (https://david.ncifcrt.gov/). Gene set enrichment analysis was conducted using the R package clusterProfiler (Yu et al., 2012), enrichplot (https://bioconductor.org/packages/release/bioc/html/enrichplot.html), and pathview (Luo and Brouwer, 2013), with *Arabidopsis thaliana* gene sets, because the functional information of many genes in fennel are still unknown.

### Fennel extracts

Plant extracts were collected from fennel leaves after the drying process according to a previously described method (Kao et al., 2011). The dried leaves and stems (0.5 g) were extracted with 25 mL 99% methanol (Daejung, Korea) at 58°C for 24 h using a shaking incubator (ED-SI300R, HYSC, Korea), and the supernatants were then collected from plant extracts using a centrifuge (Allegra X-30R Centrifuge, BECKMAN COULTER, USA) at 1300 RCF (×*g*) for 10 min. Fennel methanol extract was used to measure antioxidant activity.

### Total phenolic content

TPC was measured using the Folin-Ciocalteu method (Slinkard and Singleton, 1977; Odabasoglu et al., 2004) with some modifications. A mixture of 50 μL fennel extract and 50 μL Folin-Ciocalteu’s phenol reagent (Sigma-Aldrich, Burlington, MA, USA) was incubated for 6 min in the dark at room temperature of around 25 to 27°C. The reactant was mixed with 0.5 mL of 7% sodium carbonate solution (Daejung) and 250 μL of distilled water, and then incubated for 90 min under dark conditions. The absorbance of the solution was measured at 760 nm using a spectrometer (OPTIZEN POP, KLAB, Korea). The standard curve equation was generated using gallic acid (Daejung) as the standard at different concentrations, and used for the quantification of TPC (**Figure S3**).

### Total flavonoid content

TFC was measured using a previously described method (Zhishen et al., 1999) with some modifications. A mixture of 50 μL fennel extract and 100 μL 5% sodium nitrate solution was incubated for 6 min at room temperature of around 25 to 27°C, and 150 μL of 10% aluminum chloride solution (Daejung) was added. After 5 min, 200 μL of 1 M sodium hydroxide solution (Daejung) was added, and the mixture was incubated for 1 h at 37°C. The absorbance of the solution was measured at 510 nm using a spectrophotometer. A TFC standard curve was obtained using quercetin (Sigma-Aldrich) for fennel methanol extract (**Figure S3**). The calibration curve was generated based on different concentrations of quercetin.

### Free radical scavenging (DPPH) activity

Free radical scavenging (DPPH) was estimated according to a procedure described previously (Brand-Williams et al., 1995) with some modifications. A mixture of 100 μL plant extracts and 900 μL 0.1 mM DPPH solution was incubated for 10 min at room temperature of around 25 to 27°C, and the absorbance of the solution was then measured at 515 nm using a spectrometer. Gallic acid was used as the standard, and the standard curve was generated as described above (**Figure S3**). The control absorbance was measured using a 0.1 mM DPPH solution. DPPH scavenging activity was calculated for each treatment as follows:


$$Inhibition\;\left(\%\right)=\left(1-\;\frac{sample\;absorbance}{control\;absrobance}\right)\times 100$$

### Nitrate-scavenging activity

Nitrate scavenging activity was estimated using a method described previously (Kato et al., 1987) with some modifications. A mixture of 50 μL of plant extract, 50 μL of sodium nitrite solution (Daejung), and 300 μL of 0.1 N HCl (pH 1.2) was incubated for 1 h at 37°C, and 1 mL of 2% acetic acid solution and 100 μL of Griess reagent (Sigma-Aldrich) were then added. After incubation for 15 min at room temperature of around 25 to 27°C, the absorbance of the solution was measured at 520 nm using a spectrometer. Nitrate scavenging activity was calculated as follows for each treatment:


$$Nitrate\;scavenging\;activity\;\left(\%\right)=\left(1-\;\frac{\left(A-C\right)}{B}\right)\;\times 100$$

where *A* represents the absorbance of the sodium nitrite solution containing Griess reagent and plant extracts, *B* represents the absorbance of the sodium nitrite solution containing Griess reagent, and *C* represents the absorbance of the sodium nitrite solution containing distilled water and plant extracts.

### Ferric reducing antioxidant power assay

The FRAP assay was performed as described previously (Benzie and Strain, 1996) with some modifications. The FRAP reagent was composed of 300 mM acetate buffer (pH 3.6; Sigma-Aldrich), 10 mM 2,4,6-tripyridyl-s-trianzine (Sigma-Aldrich), and 20 mM ferrous chloride (Sigma-Aldrich) at a 10:1:1 (v/v/v) ratio. Plant extracts (30 μL) were mixed with 900 μL of FRAP reagent shortly before the FRAP assay was performed. After 10 min at 37°C, the solution absorbance was measured at 590 nm using a spectrometer. Gallic acid was used as the standard, and the standard curve was plotted as described above. The FRAP was quantified based on the standard curve equation of Gallic acid (**Figure S3**).

### Reducing power assay

The reducing power assay was performed as described previously (Oyaizu, 1986), with some modifications. A mixture of 300 μL of plant extract, 300 μL of 200 mM phosphate buffer (pH 6.6; Sigma-Aldrich), and 300 μL of 1% potassium ferricyanide (Sigma-Aldrich) was incubated in a water bath (JSWB-22(T), JSR, Korea) for 20 min at 50°C. The reactants were mixed with 300 μL of 10% trichloroacetic acid (Sigma-Aldrich) solution and then centrifuged at 13,000 rpm using a Centrifuge 5415 C machine (Eppendorf, Germany). Ferric chloride (300 μL; Sigma-Aldrich) was added to 300 μL of the supernatant, and the absorbance of the solution was measured at 700 nm using a spectrometer.

### Statistical analysis

The CO_2_-treated groups (400 or 800 ppm) were analyzed to determine significant differences using two-tailed Student’s *t*-test. The statistical data were analyzed using MS Excel 2016 (Microsoft Corp., Redmond, WA, USA). The results are presented as the mean along with the standard deviation from three independent biological replications. The equations and R-squared value of standard curves were determined using MS Excel 2016 (Microsoft).

## Results

### Effects of eCO2 concentration on fennel growth

**Figure 1:**
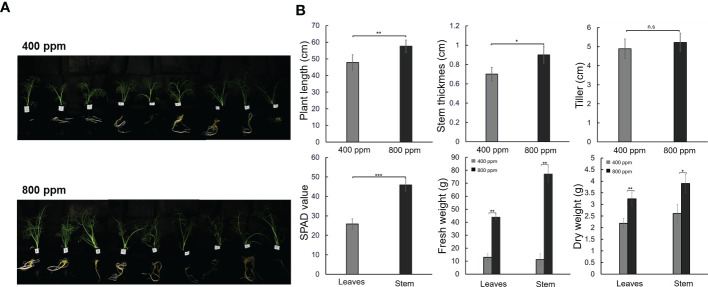
Growth characteristics in fennel under different CO_2_ treatments (400 and 800 ppm). **(A)** Phenotypes of fennel four weeks. **(B)** Growth differences is fennel over five weeks between CO_2_ treatments. The values represent the mean ± standard deviation (n = 9). Statistical significance is based on two-tailed Student’s *t*-test (*p < 0.05, **p < 0.01, ***p < 0.001, n.s, non-significance).

To evaluate the effects of eCO2 on plant growth, we observed the phenotypic changes in fennels under 400 or 800 ppm CO2 conditions (**Figure 1**). The fennels showed distinct growth characteristics five weeks after planting (**Figure 1A**). The relatively high CO_2_ concentrations promoted shoot and root development, with fennels exhibiting increased shoot length and root phenotype. There were significant differences in plant length, stem diameter, SPAD value, and tissue weight in fennel plants between the 400 and 800 ppm groups. However, there were no significant differences in number of tillers (**Figure 1B**). In particular, the SPAD value in the 800 ppm group was double that in the 400 ppm group (45.87 vs. 25.85, respectively). The fresh weights of leaves and stem tissues were largely higher in the 800 ppm group, at 43.86 and 77.09 g, respectively, when compared with 12.97 and 11.24 g, respectively, in the 400 ppm group. Plant length was significantly different between the 400 and 800 ppm groups (49.81 vs. 57.64 cm, respectively). Additionally, the dry weights of tissues increased significantly in fennels under the 800 ppm CO_2_ condition. However, the differences in plant tissues between the fresh and dry weights decreased, indicating relatively high contents in the 800 ppm group. The results indicate that relatively high CO_2_ concentrations promoted plant growth and leaf chlorophyll concentrations.

### Effects of CO_2_ supply and light on hydroponics environment

**Figure 2:**
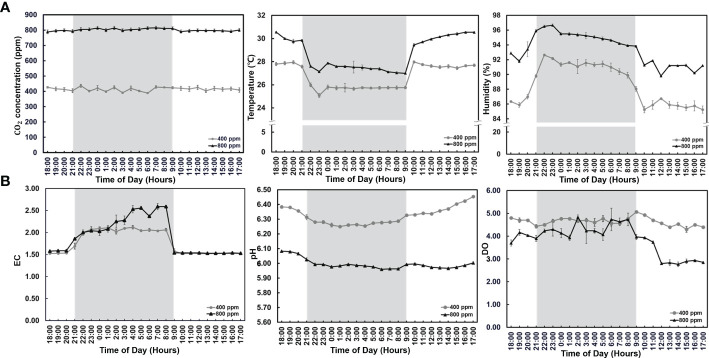
Environmental factors and nutrient solution characteristics after CO_2_ treatment for five weeks. **(A)** Environmental factors (CO_2_ concentration, and atmospheric temperature and humidity). **(B)** Nutrient solution (electrical conductivity, pH, and dissolved oxygen). The background colors represent the different light mean ± standard deviation (n = 9). The line symbols indicate the CO_2_ treatment (circle; 400 ppm, triangle; 800 ppm).

To determine the effect of CO_2_ supply in hydroponics under different light conditions, we observed distinct changes in environmental factors (temperature and humidity) and nutrient solution composition (EC, pH, and DO) in the fennel plants for five weeks after planting (**Figure 2**). The CO_2_ concentrations in each sealed greenhouse were maintained using a customized CO_2_ system (**Figure 2**). In days without light, the temperature and humidity increased in the greenhouse under the 800 ppm conditions when compared with those under the 400 ppm condition. Furthermore, lower temperature and higher humidity were observed in the greenhouses under the dark condition than under the light condition. The EC was relatively high in late dark conditions for hydroponics (light condition: 1.53 vs. 1.54 mS for the 400 and 800 ppm groups, respectively, dark condition 2 vs. 2.2 mS for the 400 and 800 ppm groups, respectively). In addition, the pH in the nutrient solution for the 800 ppm group was higher than that in the 400 ppm group. The DO decreased under light conditions in the 800 ppm group when compared with those in the 400 ppm group. These results indicate that the environment and nutrient solutions were changed considerably in fennel under the dark condition owing to distinct photosynthesis and transpiration characteristics based on differences in light intensity and eCO2.

### *De novo* transcriptome analysis

To survey transcriptional changes in response to different CO_2_ concentrations, we conducted RNA sequencing analysis in fennels under the 400 and 800 ppm treatments. We assembled a total of 174,266,880 bases with an average contig length of 1250, and 139,352 transcripts were detected (data not shown). The 31,737 assembled contigs were analyzed to determine the DEGs, and the assembled contigs were highly correlated in each experiment under the 400 or 800 ppm conditions (**Figure S2A**). A total of 4,965 DEGs, including 1,776 downregulated and 3,189 upregulated genes, were detected in fennels cultivated under the different CO_2_ concentrations (**Figure S2B**).

**Figure 3:**
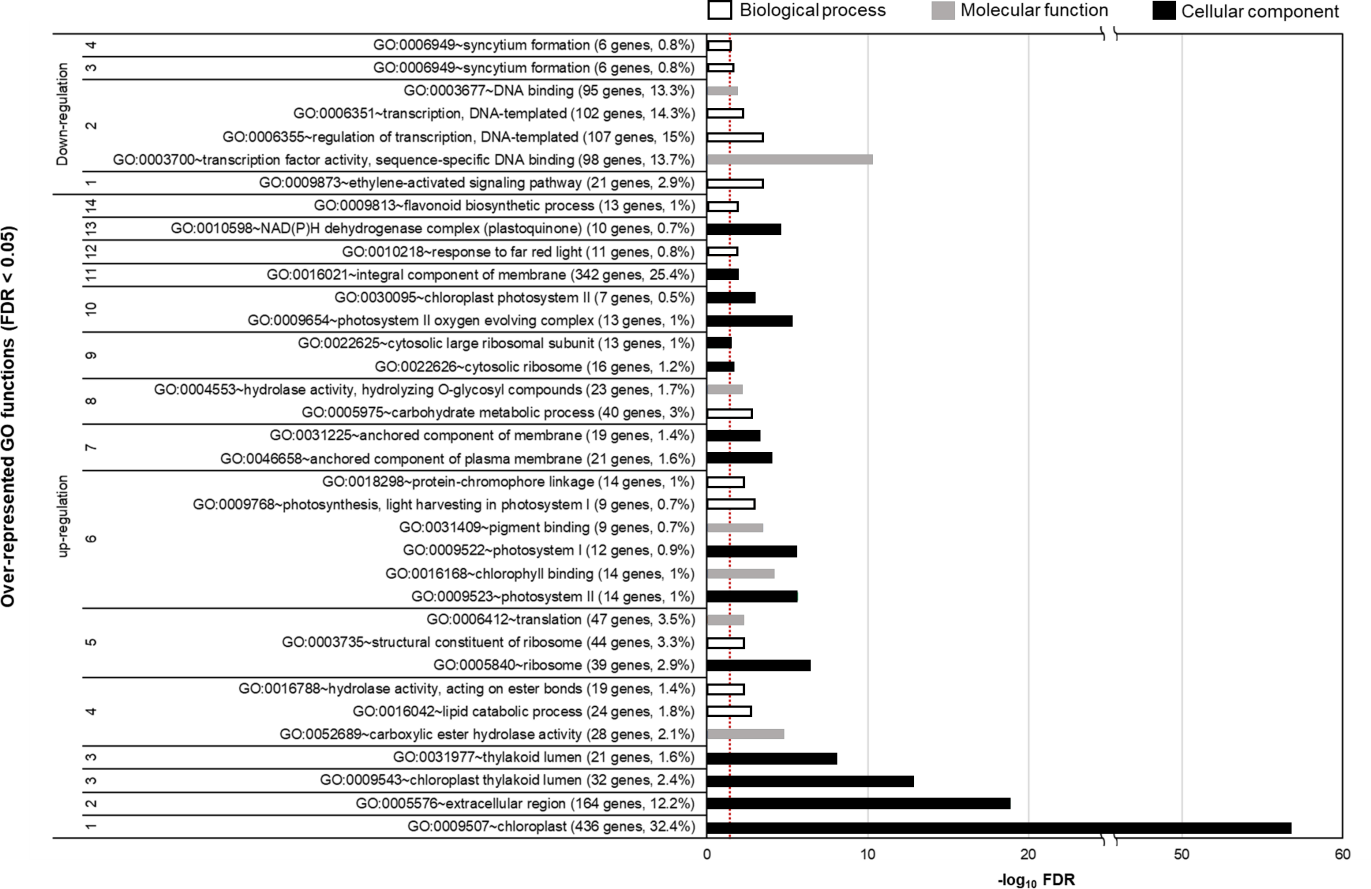
Over-represented Gene Ontology (GO) functions of CO_2_-responsive genes (false discovery rate [FDR]< 0.05). The over-represented GO functions were determined by DAVID functional annotation tool (https://david.ncifcrf.gov). The parenthesis indicates the number and percentage of genes involved in each Go function. The bar color represents the different GO functions. The dashed line indicates the threshold (-log_10_0.05) for statistical significance.

To determine the significantly altered gene functions of DEGs, we conducted a functional enrichment analysis of the upregulated and downregulated genes in the 800 ppm group (**Figure 3**). We detected 17 groups (14 and 3 groups for the upregulated and downregulated DEGs, respectively) of overrepresented Gene Ontology terms (FDR< 0.05), including cellular component, biological process, and molecular function. Many groups were associated with photosynthetic mechanisms in chloroplasts, thylakoid lumen-related cellular component, photosystem-related functions, and response to light. In addition, the DEGs involved in lipid catabolic process, translation-related functions, hydrolase activity, and flavonoid biosynthetic process were significantly upregulated in fennels under the 800 ppm condition. However, 3 ethylene-activated signaling pathway, transcription, and syncytium formation were downregulated in the 800 ppm group.

**Figure 4:**
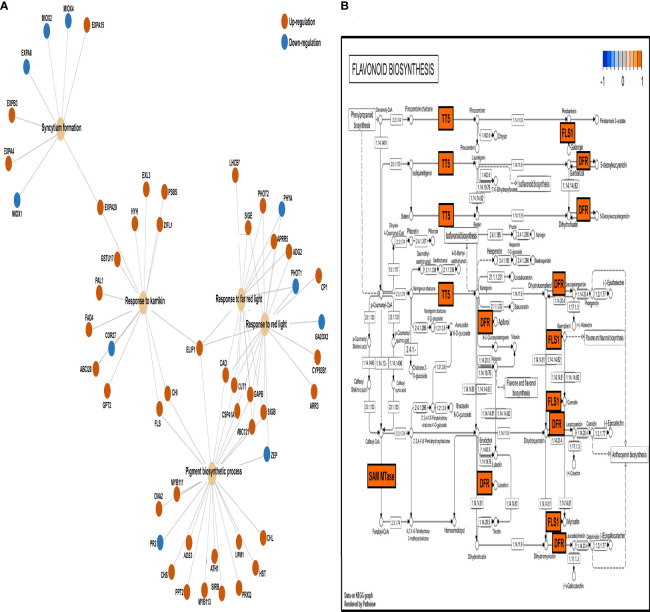
Gene set enrichment analysis of CO_2_-responsive genes. **(A)** The connection of genes among the prominent Gene Ontology (GO) functions based on enrich GO function. The colored circles represent the different regulations of genes with log fold-change (FC) values (red; upregulation, green; downregulation). **(B)** Expression patterns of differentially expressed genes (DEGs) in the flavonoid biosynthesis pathway based on Kyoto Encyclopedia of Genes and Genomes (KEGG) database (https://www.genome.jp/kegg/). The DEGs were mapped using the pathview package in R. The colored boxes represent the different gene regulation characteristics based on log FC value (red; upregulation, green; down regulation) (21.1.104 > S-adenosyl-L-methionine-dependent methyltransferases [SAM MTase], 5.5.1.6 > transparent testa 5 [TT5], 1.1420.6 > flavonoid synthase 1 [FLS1], 1.1.1.219 > dihydroflavonol 4-reductase [DFR], 1.1.1234 > DFR).

To detect overrepresented pathways in a priori-defined gene set, we conducted gene set enrichment analysis (**Figure 4**) and determined the connections and regulation of genes involved in prominent Gene Ontology biological processes (pigment biosynthetic process, response to far-red/red light, response to Karrikin, and syncytium formation) (**Figure 4A**). The DEGs of the four pathways were mainly upregulated in the 800 ppm group, in which 39 DEGs were upregulated and six were downregulated. The eight DEGs related to syncytium formation showed four upregulated and four downregulated genes. To determine whether relatively high CO_2_ concentration (800 ppm) affected the biomass increase in fennels, we observed the gene expression patterns under flavonoid biosynthesis (**Figure 4B**). Notably, several genes involved in the flavonoid biosynthetic pathway were upregulated, whereas no genes were downregulated, suggesting flavonoid content increase under 800 ppm.

### Antioxidant activity

**Figure 5:**
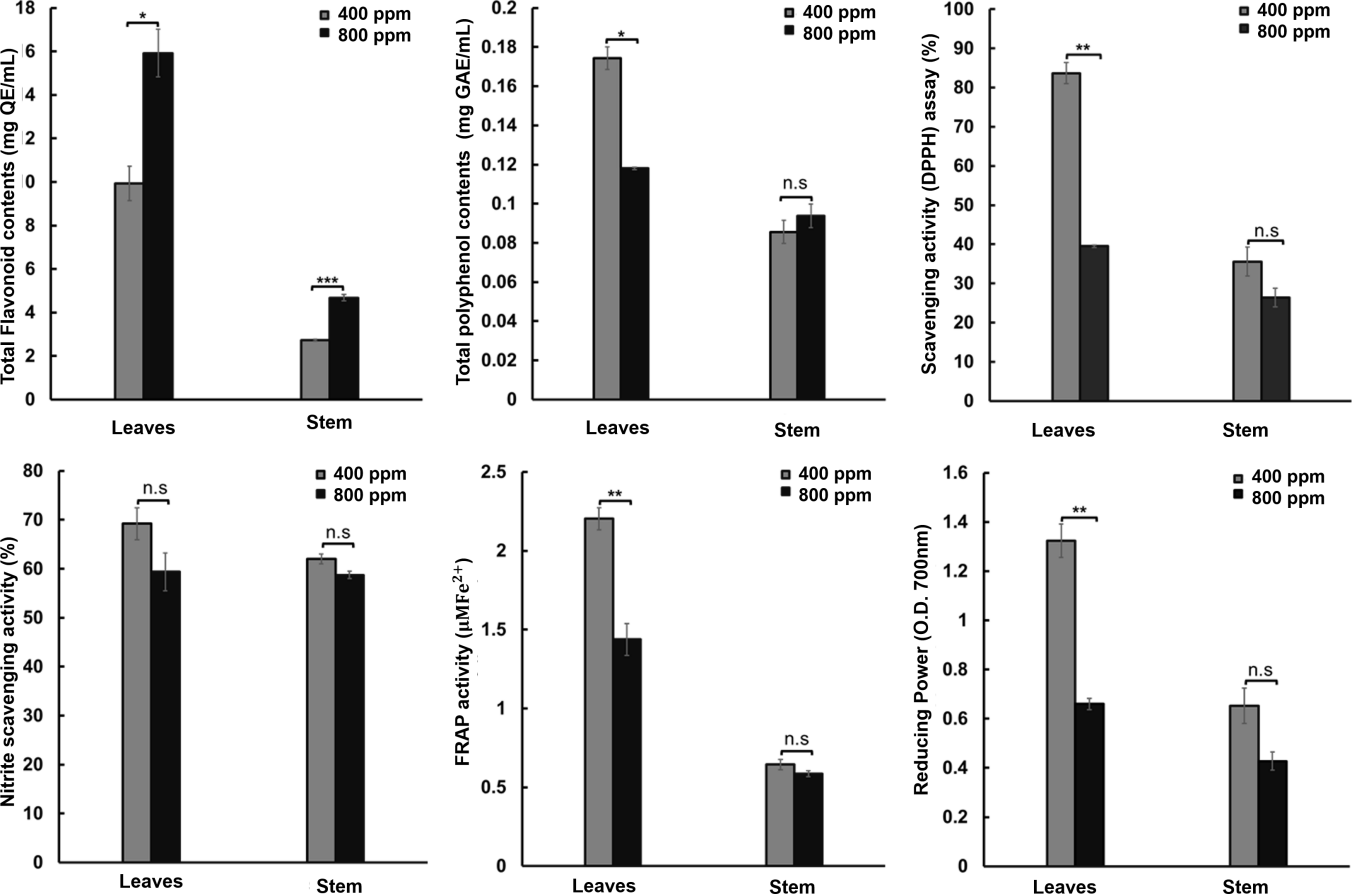
Antioxidant activity (Total flavonoid contents, total polyphenol contents, free radical scavenging [DPPH] activity, nitrite scavenging activity, ferric reducing antioxidant power [FRAP] activity, and reducing power activity) of fennel extract following treatment with 400 and 800 ppm CO_2_. The values represent the mean ± standard deviation (n = 9). Statistical significance is based on two-tailed Student’s t-test (*p < 0.05, **p < 0.01, ***p < 0.001, n.s, non-significance).

To confirm the possibility of biomass increase under the 800 ppm condition, we conducted antioxidant activity assays for TPC, TFC, DPPH, nitrite-scavenging, FRAP, and reducing power in the leaves and stems of fennels in the 400 and 800 ppm groups (**Figure 5**). We confirmed that TFC was significantly increased in both the leaves and stems of fennel, confirming the upregulation of the flavonoid pathway-related DEGs. The TFC was 9.93 vs. 15.92 mg QE/mL for the 400 and 800 ppm groups in the leaves and 2.73 vs. 4.86 mg QE/mL for the 400 and 800 ppm groups, respectively, in the stems. However, the rest of the antioxidant activity assays showed significantly decreased activity in the leaves of the fennels in the 800 ppm group; compared to the 400 ppm group, the antioxidant activities in the 800 ppm group were decreased by 35.29% in the TPC assay (0.17 vs. 0.11 Mg GAE/mL for the 400 and 800 ppm groups, respectively), 50.76% in the reducing power assay (1.32 vs. 0.65 O.D. in the 400 and 800 ppm groups, respectively), 52.8% in the DPPH scavenging activity assay (88.66% vs. 39.48% in 400 and 800 ppm groups, respectively), and 35% in the FRAP assay (2.2 O.D. vs. 1.43 O.D. in the 400 and 800 ppm groups, respectively). Although the plants in the 800 ppm group exhibited slightly increased nitrite-scavenging activity, no significant differences were detected between the stems and leaves of the fennels.

### Growth and antioxidant activity of fennel under different light intensity conditions

**Figure 6:**
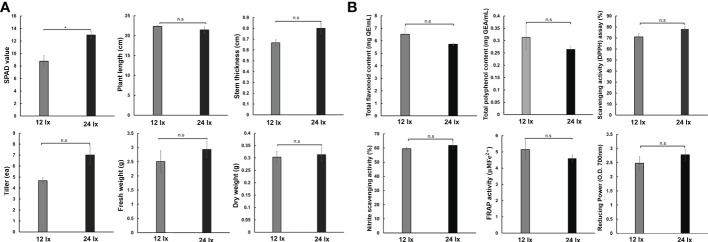
Fennel growth survey under different light conditions (12 lx and 24 lx) and no CO_2_ treatment. **(A)** Growth characteristics in fennel among different treatments. **(B)** Contents of antioxidant substances. The values represent the mean ± standard deviation (n = 9). Statistical significance is based on two-tailed Student’s *t*-test (*p < 0.05, n.s, non-significance).

As the plants grew in the hydroponics systems, the fennels were exposed to strong light intensity because of the limited spatial extent between artificial light and growth space. The length of fennel increased under the 800 ppm CO_2_ condition, as mentioned above (**Figure 1B**). Therefore, we investigated whether the improved plant traits were more strongly affected by light intensity or eCO_2_ concentrations, by observing plant growth and antioxidant activities in fennel under different light intensities (12 lx and 24 lx) (**Figure 6**). When we observed changes in environmental factors and nutrient solution composition, there were no notable differences between 12 lx and 24 lx (**Figure S4**). We confirmed increased humidity, EC, and DO, and decreased temperature and pH in the greenhouse under dark condition regardless of light intensity. For a day, the distinct changes between light and dark conditions was similar to eCO_2_. Furthermore, we examined six plant traits, including SPAD, plant length, stem thickness, tiller number, and fresh and dry weights of fennel under the same concentrations (230 ppm) of CO2 (**Figure 6A**). We found significantly increased (p< 0.001) SPAD units in fennel under the 24 lx conditions than under the 12 lx conditions. No significant differences in other plant traits were observed in fennel under the two light conditions. Four weeks after planting, the average lengths of the plants were 32 cm and 48 cm in the 400 and 800 ppm groups, respectively (data not shown). However, the fennel had short plant lengths under different light conditions (22.33 cm and 21.43 cm under the 12 lx and 24 lx conditions, respectively) because of the low CO_2_ concentrations in the growth room. During the same period of plant growth (four weeks after planting), unlike eCO_2_ concentration, the different light conditions did not affect the lengths of the fennel plants or the antioxidant activities of the plant extracts (**Figure 6B**). The results suggest that CO_2_ has a stronger effect on plant improvement than light intensity.

## Discussion

Previous studies have reported that eCO_2_ concentrations affect plant physiology (Eamus et al., 1995; Taylor et al., 2001); however, the growth responses of fennel under different CO_2_ conditions are still unknown. In the present study, we found that under eCO_2_ concentrations, the fennel plants exhibited distinct growth responses, including increases in plant length, stem diameter, tiller number, and SPAD value. The results are consistent with the results of previous studies reporting that several plants, such as *Zea mays*, sugarcane, and *Sorghum bicolor*, exhibit improved growth responses in terms of leaf area, plant height, and total mass under high CO_2_ conditions (De Souza et al., 2008; Sicher and Barnaby, 2012; Adishesha et al., 2017; Khanboluki et al., 2018; Huang et al., 2019; Mina et al., 2019).

Leaf growth is enhanced under CO_2_ conditions through cell expansion (Ranasinghe and Taylor, 1996). Under eCO_2_ concentrations, leaf weight increased in millet, lambsquarters, and soybean (Miri et al., 2012). In addition, high CO_2_ concentrations resulted in increased chlorophyll contents in many plants, including millet, soybean, pigweed, and lambsquarters (Miri et al., 2012). High CO_2_ concentrations generally increase the photosynthetic parameters of plants (Hao et al., 2013; Wang et al., 2018). We also observed increased SPAD value in fennel under relatively high CO_2_ concentrations. The result was partially supported by the increased concentrations of leaf chlorophyll under eCO_2_.

For understanding the physiology of stomata and gas exchange, it is important to elucidate the stomatal responses of vegetation to atmospheric CO_2_ concentrations. eCO_2_ concentrations lead to reduction in stomatal density and leaf-level transpiration (Lawson et al., 2003; Haworth et al., 2013; Kirschbaum and McMillan, 2018). In addition, a decrease in transpiration rate negatively affects relative humidity during plant growth (Mishra and Pradhan, 1972). In our study, we found that the temperature and humidity generally increased in plastic film-covered greenhouses under 800 ppm CO_2_ regardless of photoperiod, suggesting that the humidity decreased under relatively low CO_2_ concentrations. The temperature was increased in the greenhouse for plant growth until beginning of crop maturation, whereas the humidity was decreased (Mendoza-Pérez et al., 2018). Therefore, the results suggest that the relatively high degrees of temperature and humidity were caused by improved plant growth and/or stomatal reduction under high eCO_2_ concentrations. Furthermore, we observed distinct temperature and humidity characteristics between the light and dark conditions. Similarly, (Hein et al., 2019) humidity was decreased in response to an increase in temperature and vapor pressure deficit value. The results suggest that humidity was increased in the greenhouses by the decreased temperature under dark condition.

EC is a salt/electrolyte concentration index in nutrient solution and is related to the number of ions available for plant roots (Nemali and van Iersel, 2004). High EC increases the chlorophyll, leaf water, ascorbic acid, and crude protein contents in pakchoi (*Brassica campestris* L. ssp. Chinensis) under hydroponic conditions (Ding et al., 2018). In addition, EC in nutrient solution varies under different light conditions, with relative EC increasing as the night time increases (Albornoz et al., 2014). Similarly, we observed increased SPAD values in fennel under dark conditions. Moreover, elevated atmospheric CO_2_ concentrations reduce pH in the ocean because CO_2_ is soluble in water (Feely et al., 2006). Similarly, we observed decreased pH in the nutrient solution under relatively high CO_2_. In addition, substrate-EC negatively interacts with substrate-pH in the growing medium in greenhouses (Dickson, R., Fisher, P., 2017. High pH is often caused by excess leaching. Greenhouse management.). In the present study, we also observed slightly lower pH caused by increased EC in the nutrient solution under dark conditions compared to under light conditions. Moreover, DO showed a relatively high concentration during the change in light conditions from light to dark due to photosynthesis-induced oxygen accumulation. The DO is generally the highest at sunset during the day and gradually decreases at night. However, in our study, the DO did not decrease in the hydroponics because of the air supply system.

In addition, our RNA sequencing analysis revealed transcriptional changes in genes associated with photosynthesis and antioxidants in fennel under relatively high CO_2_ concentrations, many genes related to photosynthetic responses were upregulated. The results are consistent with those of the study by Zhao et al. (Zhao et al., 2019), who reported that many genes involved in photosystems I and II were significantly upregulated in grapes under increased CO_2_ concentrations. In addition, we identified upregulated DEGs in the Karrikin signaling pathways. Karrikins are produced following burning of plant material, in smoke, and act as plant growth regulators (Chiwocha et al., 2009; Flematti et al., 2015). In particular, Karrikins promote plant development and germination because they function similar to the plant hormone strigolactone (Andreo-Jimenez et al., 2015). Karrikins also improve net photosynthesis rate and chlorophyll content in carrots as a result of increased stomatal conductance and high intercellular CO_2_ concentrations (Akeel et al., 2019). Therefore, the Karrikin genes upregulated by eCO_2_ may promote fennel growth. Additionally, four genes (transparent testa 5, S-adenosyl-L-methionine-dependent methyltransferases, dihydroflavonol 4-reductase, and flavonoid synthase 1) involved in flavonoid biosynthesis were upregulated in the plants in the 800-ppm group. The induced transparent testa 5 mRNA expression led to an increase in TFC in Arabidopsis transgenic lines (Rao et al., 2019). S-adenosyl-L-methionine-dependent methyltransferases reportedly play important roles in flavonoid-related metabolic pathways (Joshi and Chiang, 1998). The high expression levels of dihydroflavonol 4-reductase, a key enzyme involved in anthocyanin biosynthesis, resulted in an increase in the TFC in *Camellia* transgenic lines (Jiang et al., 2020). Flavonoid synthase overexpression led to flavonoid accumulation as well as the suppression of anthocyanin synthesis in Crabapples (Tian et al., 2015). On the basis of previous studies and the results of the present study, we hypothesize that CO_2_ supply could increase flavonoid contents in fennel by altering gene expression levels.

In the present study, we investigated the effect of eCO_2_ on the antioxidant activity of fennel and found that total polyphenol content, scavenging activity, FRAP, and reducing power were decreased significantly in equal quantities of fennel leaves and stem under 800 ppm CO_2_ conditions. However, the dry weight of the plants in the 800 ppm group was approximately 4.9 times that of the plants in the 400 ppm group; therefore, considering that the antioxidant activity was assayed in equal amounts of plant extracts and the improved plant growth in the 800 ppm group, the antioxidant activities were similar between the groups under the two CO_2_ conditions. Previous studies have reported that eCO_2_ leads to improved antioxidant accumulation in vegetables; antioxidants such as total phenols, total flavonoids, and ascorbic acid were increased by CO_2_ supply (Dong et al., 2018). However, TPC decreased in *Brassica rapa* and *Gynostemma pentaphyllum* under high CO_2_ concentrations (Karowe and Grubb, 2011; Chang et al., 2016). CO_2_ reportedly affects the content of flavonoid and phenolic compounds in young Malaysian ginger (Ghasemzadeh et al., 2010). We confirmed that TFC was significantly increased in both the flavonoid pathway-related DEGs. Furthermore, light intensity affects antioxidant system activity in basil and lettuce; for example, under strong light intensity, the DPPH, ABTS, and FRAP showed decreased activities in basil, and increased activity in lettuce (Sutulienė et al., 2022). Although the effects of light intensity on antioxidant activity have been reported in other plants, we did not observe significant differences in fennel under different light conditions, unlike in the case of CO_2_. For example, the fennel showed no significant differences in plant growth and antioxidant activity, whereas significant differences were observed between the 400 ppm and 800 ppm groups. The results suggest relatively stronger effects of eCO_2_ concentration on plant growth and antioxidant activities than light intensity in the 12 and 24 lx range. The results suggest that eCO_2_ concentration increases plant growth and antioxidant contents in fennel in agricultural settings.

## Conclusions

We analyzed the effects of eCO_2_ on plant growth and antioxidant activity in hydroponic systems. Several traits related to plant growth, including plant length, leaf length, SPAD value, and fresh and dry weights were improved in fennel under five weeks of relatively eCO_2_. In addition, changes in the environment and nutrient solutions were observed in hydroponics under different light conditions. The pH decreased in the nutrient solution under dark and eCO_2_ conditions, as a result of increased EC. In the transcriptome analysis, we detected eCO_2_-responsive genes involved in response to red/far-red light, pigment biosynthetic process, and response to Karrikin, and observed that several genes were upregulated in the flavonoid biosynthesis pathway. eCO_2_ increased amounts of total flavonoids in fennel, whereas five antioxidants, excluding, flavonoids, were decreased. We investigated whether the improved plant traits were more strongly affected by light intensity or eCO_2_. Subsequently, we observed different effects on fennel between eCO_2_ and light intensity for the same plant growth period. The increased eCO_2_ concentration led to significant changes in plant growth and antioxidants, while the increased light intensity did not significantly affect the growth or antioxidant activities of fennel. This result suggests that the eCO_2_ had relatively strong effects on plant growth compared with light intensity. Our findings can provide a basis for improving the agricultural traits of fennel, which is a natural source of functional materials under eCO_2_.

## Data availability statement

The data presented in the study are deposited in the NCBI repository, accession number GSE218441.

## Author contributions

NJ and SH designed the study. NJ, JL, JB, HP, JR and SH data collected and analyzed. NJ and SH wrote the manuscript. All authors contributed to the article and approved the submitted version.

## Supplementary material

Supplementary Figure 1 Constant CO_2_ concentration of 400 or 800 ppm controlled using Arduino UNO R2 with an ATMEGA-489 microcontroller. **(A)** The CO_2_ sensor has a range of measurement of 0 to 5,000 ppm and an error ± 30 ppm. **(B)** CO_2_ 20-L aluminum cylinder tank with gas regulator.

Supplementary Figure 2 RNA sequencing of CO_2_-treated fennel at 400 ppm or 800 ppm. **(A)** The hierarchical clustering of differentially expressed genes in the replicated samples. **(B)** The number of up- or downregulated genes.

Supplementary Figure 3 The standard curves of total phenol, total flavonoid, DPPH, and ferric reducing antioxidant power.

Supplementary Figure 4 Environmental factors and nutrient solution characteristics after light intensity treatment for five weeks. **(A)** Environmental factors (CO_2_ concentration, and atmospheric temperature and humidity). **(B)** Nutrient solution (electrical conductivity, pH, dissolved oxygen). The background colors represent the different light mean ± standard deviation (n = 9). The line symbols indicate the light intensity treatment (circle; 12 lx, triangle; 24 lx).
