# Supplementary figures and images for: Elevated CO2 concentration induces changes in plant growth, transcriptome, and antioxidant activity in fennel (Foeniculum vulgare Mill.)
Source: Front Plant Sci. 2022 Dec 9;13:1067713. doi: 10.3389/fpls.2022.1067713 (PMC9780672; doi:10.3389/fpls.2022.1067713)

**A**

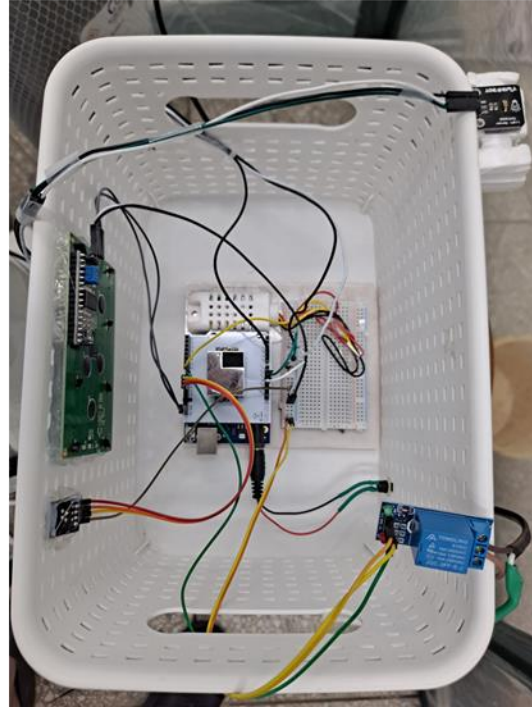

**B**

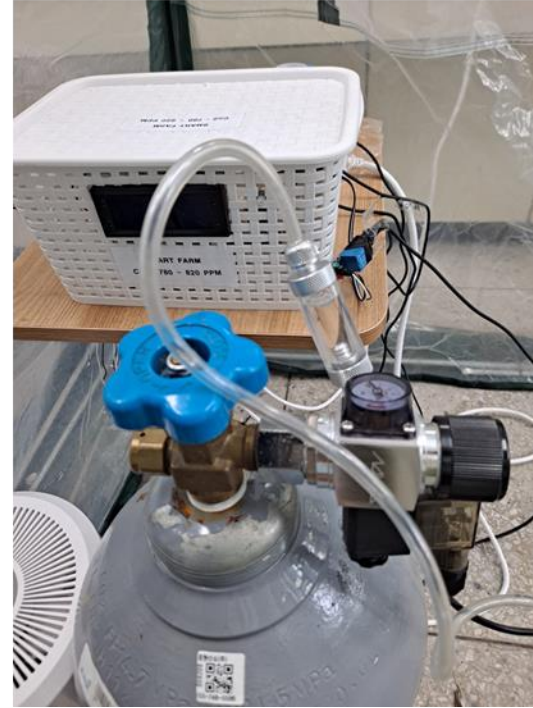

Jo et al., Fig. S1

**A**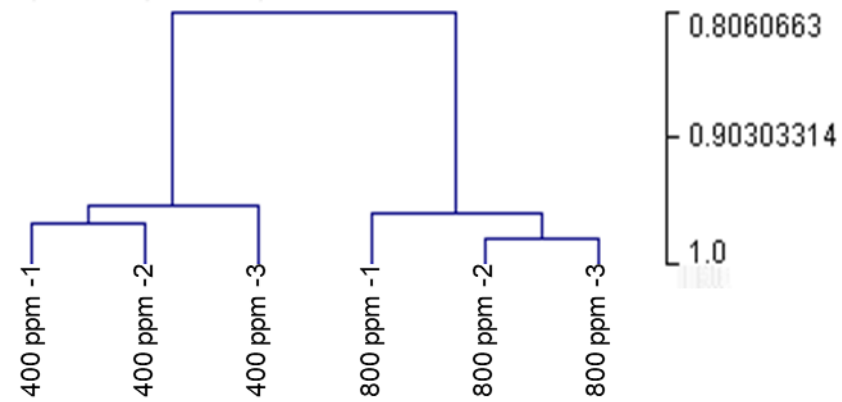**B**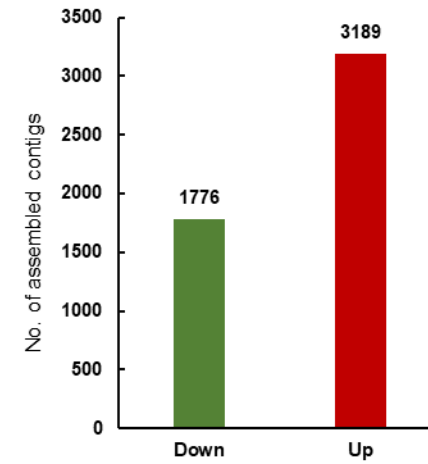

Jo et al., Fig. S2

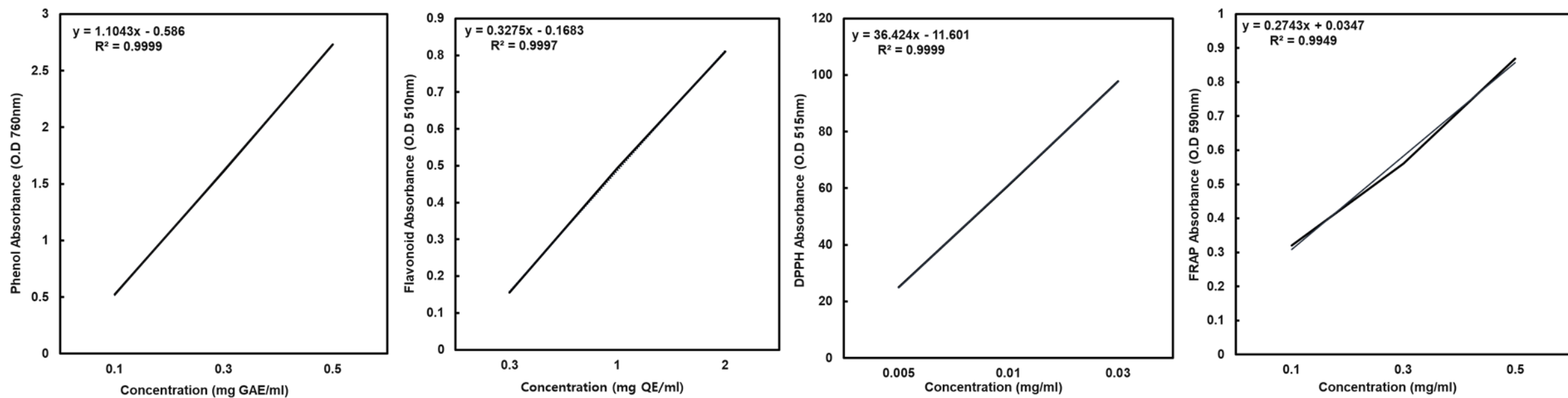

Jo et al., Fig. S3

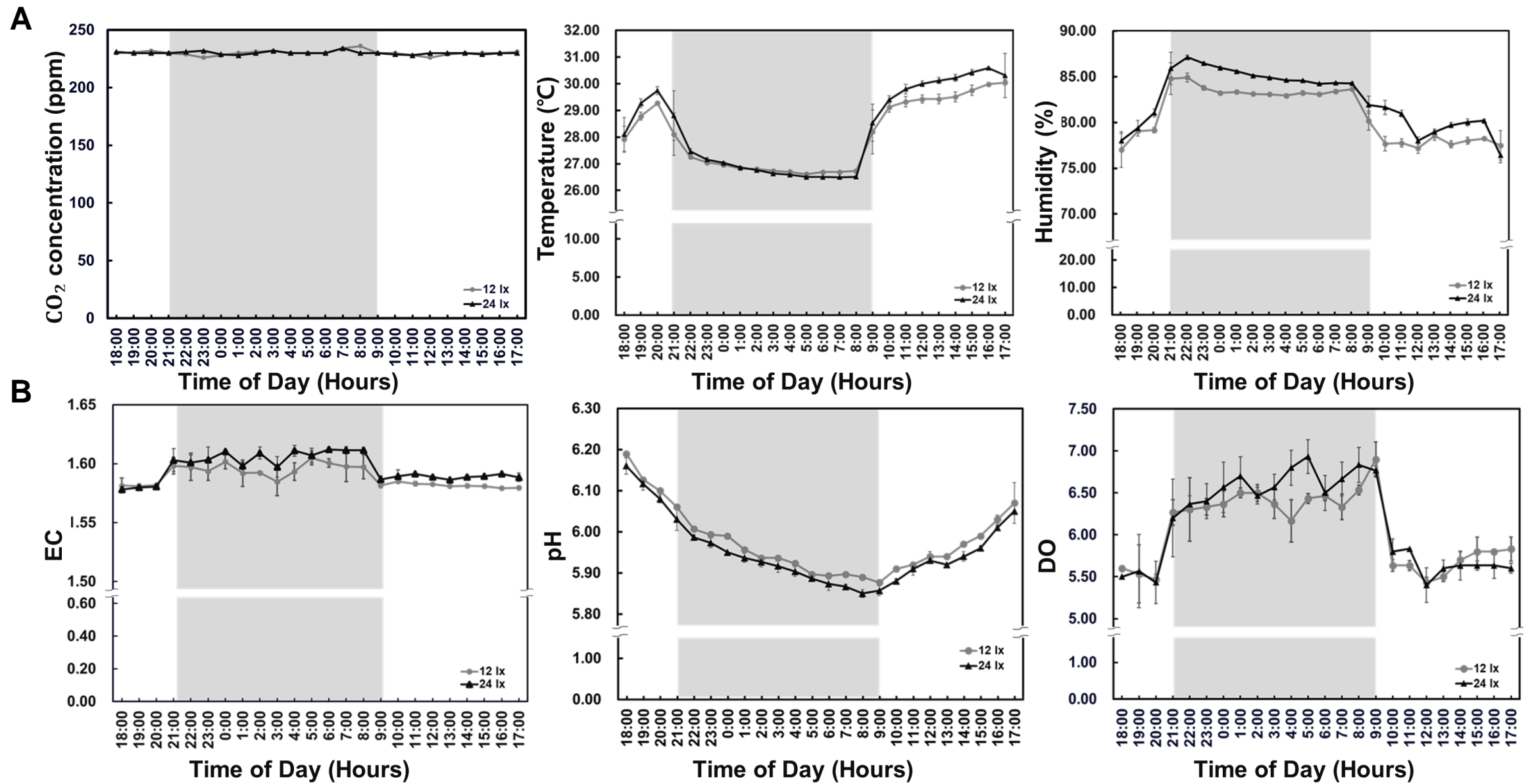

Jo et al., Fig. S4

Supplement: Supplementary Figure 1 — Constant CO2 concentration of 400 or 800 ppm controlled using Arduino UNO R2 with an ATMEGA-489 microcontroller. (A) The CO2 sensor has a range of measurement of 0 to 5,000 ppm and an error ± 30 ppm. (B) CO2 20-L aluminum cylinder tank with gas regulator. [file DataSheet_1.pdf]
